# A Gene-Based Analysis of Variants in the Serum/Glucocorticoid Regulated Kinase (SGK) Genes with Blood Pressure Responses to Sodium Intake: The GenSalt Study

**DOI:** 10.1371/journal.pone.0098432

**Published:** 2014-05-30

**Authors:** Changwei Li, Xueli Yang, Jiang He, James E. Hixson, Dongfeng Gu, Dabeeru C. Rao, Lawrence C. Shimmin, Jianfeng Huang, Charles C. Gu, Jichun Chen, Jianxin Li, Tanika N. Kelly

**Affiliations:** 1 Department of Epidemiology, Tulane University School of Public Health and Tropical Medicine, New Orleans, Louisiana, United States of America; 2 State Key Laboratory of Cardiovascular Disease, Fuwai Hospital, National Center of Cardiovascular Diseases, Chinese Academy of Medical Sciences and Peking Union Medical College, Beijing, China; 3 Department of Medicine, Tulane University School of Medicine, New Orleans, Louisiana, United States of America; 4 Department of Epidemiology, Human Genetics and Environmental Sciences, University of Texas School of Public Health, Houston, Texas, United States of America; 5 Division of Biostatistics, Washington University School of Medicine, St. Louis, Missouri, United States of America; Osaka University Graduate School of Medicine, Japan

## Abstract

**Background:**

Serum and glucocorticoid regulated kinase (SGK) plays a critical role in the regulation of renal sodium transport. We examined the association between *SGK* genes and salt sensitivity of blood pressure (BP) using single-marker and gene-based association analysis.

**Methods:**

A 7-day low-sodium (51.3 mmol sodium/day) followed by a 7-day high-sodium intervention (307.8 mmol sodium/day) was conducted among 1,906 Chinese participants. BP measurements were obtained at baseline and each intervention using a random-zero sphygmomanometer. Additive associations between each SNP and salt-sensitivity phenotypes were assessed using a mixed linear regression model to account for family dependencies. Gene-based analyses were conducted using the truncated p-value method. The Bonferroni-method was used to adjust for multiple testing in all analyses.

**Results:**

In single-marker association analyses, *SGK1* marker rs2758151 was significantly associated with diastolic BP (DBP) response to high-sodium intervention (*P* = 0.0010). DBP responses (95% confidence interval) to high-sodium intervention for genotypes C/C, C/T, and T/T were 2.04 (1.57 to 2.52), 1.79 (1.42 to 2.16), and 0.85 (0.30 to 1.41) mmHg, respectively. Similar trends were observed for SBP and MAP responses although not significant (*P = *0.15 and 0.0026, respectively). In addition, gene-based analyses demonstrated significant associations between *SGK1* and SBP, DBP and MAP responses to high sodium intervention (*P = *0.0002, 0.0076, and 0.00001, respectively). Neither *SGK2* nor *SGK3* were associated with the salt-sensitivity phenotypes in single-maker or gene-based analyses.

**Conclusions:**

The current study identified association of the *SGK1* gene and BP salt-sensitivity in the Han Chinese population. Further studies are warranted to identify causal *SGK1* gene variants.

## Introduction

High salt intake has been associated with an elevated risk of high blood pressure (BP) and cardiovascular disease [1], [2]. BP responses to dietary sodium intake vary considerably among individuals, a phenomenon described as salt sensitivity of BP [3]. Previous family studies have documented a moderate to high heritability of salt sensitivity, generally ranging from 22% to 84% [4]–[6]. Likewise, linkage analyses and genetic association studies have suggested that genetic mechanisms may play a pivotal role in BP salt sensitivity [7]. The identification of genetic variants for salt sensitivity will help elucidate how genes interact with dietary factors to influence the regulation of BP.

The serum and glucocorticoid regulated kinases (SGK) play important roles in regulating Na^+^ transport in both proximal and distal elements of the kidney tubule [8], [9]. The SGK family of protein kinases have three isoforms, SGK1, SGK2 and SGK3 [9]. All of the three isoforms are highly expressed in kidney [10]–[12]. SGK1 stimulates the epithelial Na^+^ channel (ENaC), Na^+^/H^+^ exchanger 3 (NHE3) and many other renal tubular ion channels to regulate renal Na^+^ reabsorption [9], [13], [14]. SGK1 also contributes to the stimulation of mineralocorticoid-induced salt intake [14]. Recent studies showed increased salt concentration induces *SGK1* expression, and increased SGK1 subsequently enhances T_H_17 cell differentiation and promotes tissue inflammation [15]. These findings support the opinion that the intensity of immune cell infiltration in the kidney is correlated with the severity of salt-sensitive hypertension [16]. Thus the effect of SGK1 on sodium homeostasis and inflammation is expected to have an important impact on blood pressure control. Similar to SGK1, both SGK2 and SGK3 can influence NHE3 activities and potentially regulate Na^+^ transport [9]. SGK’s biological relevance combined with evidence in *vitro* and in *vivo* make the genes encoding SGK interesting candidates for genetic study of BP response to sodium intake. However, only a few studies have reported associations of variants in the *SGK* gene family with BP phenotypes in human samples [17]–[19]. None have investigated the joint effect of multiple variants within single genes, and none of them were conducted in the Chinese population. The aim of the current study was to assess the single and joint effects of genetic variants in *SGK* genes with BP salt sensitivity among Han Chinese participants of the Genetic Epidemiology Network of Salt-Sensitivity (GenSalt) study.

## Methods

### Study Population

The GenSalt study is a unique dietary feeding study examining gene-dietary sodium and potassium interaction on blood pressure among a rural Han population in north China with habitual high salt intake. Probands were defined as having mean SBP between 130 and 160 mm Hg and/or mean DBP between 85 and 100 mm Hg and no use of antihypertension medications. The probands were identified through a community-based BP screening carried out among adults aged 18–60 years in the study villages. Probands along with their spouses, siblings, and offspring were recruited as volunteers for the dietary intervention study. Detailed eligibility criteria for the probands and siblings/spouses/offspring have been presented elsewhere [20]. Briefly, individuals with stage-2 hypertension (≥160/100 mmHg), current or recent use of antihypertension medications, secondary hypertension, history of clinical CVD, diabetes, chronic kidney failure, liver disease or peptic ulcer disease requiring treatment during the previous two years, along with pregnant women, heavy alcohol drinkers, and those currently adhering to a low-sodium diet or unable to sign the informed consent were excluded from the study. The study recruited 1,906 eligible participants from 633 families. A total of 1,871 (98.2%) and 1,860 (97.6%) participants who completed the low-sodium and high-sodium dietary interventions, respectively, were included in the current analysis.

### Ethnics Statement

Institutional review boards at the Tulane University Health Sciences Center, Washington University School of Medicine, University of Texas School of Public Health, Fu Wai Hospital and Chinese National Human Genome Center at Beijing, and Chinese Academy of Medical Sciences approved the GenSalt study. Written informed consents for the baseline observation and for the intervention program were obtained from each participant.

### Dietary Intervention

After a 3 day baseline examination period, the study participants underwent a 7 day low-sodium dietary intervention (51.3-mmol of sodium/day) followed by a 7 day high-sodium dietary intervention (307.8-mmol of sodium/day). Dietary potassium intake remained unchanged during the intervention phases. Total energy intake was varied according to each participant’s baseline energy intake. All study foods were cooked without salt, and pre-packaged salt was added to the individual study participant’s meal when it was served by the study staff. To ensure compliance with the intervention program, participants were required to have their breakfast, lunch and dinner in the study kitchen under the supervision of the study staff during the entire intervention period. The study participants were also instructed to avoid consuming any foods that were not provided by study personnel. Three timed urinary specimens were collected at baseline and at the end of each intervention phase (days 5, 6 and 7) to monitor the compliance with the intervention. The results of 24-hour urinary excretion of sodium showed excellent compliance with the study diet among all participants. The respective mean (SD) of 24-hour urinary excretions at baseline, during the low-sodium intervention, and during the high-sodium intervention were 242.4 (66.7), 47.5 (16.0) and 244.3 (37.7) mmol for sodium, and 36.9 (9.6), 31.4 (7.7), and 35.7 (7.5) mmol for potassium.

### Phenotype Measurement

During the 3 days baseline examination, trained staff collected information on family structure, demographic characteristics, personal and family medical history, and lifestyle risk factors using a standard questionnaire. BP was measured 3 times at the same time each morning during the 3-day baseline and days 5, 6, and 7 during each intervention period according to a standard protocol. All BP was measured by trained and certified observers using a random zero sphygmomanometer with the participants in the sitting position after resting for 5 minutes [21]. Participants were advised to avoid alcohol, cigarette smoking, coffee/tea, and exercise for at least 30 minutes before the BP measurements. All BP observers were unaware of the dietary intervention stages. In addition, body weight and height were measured twice during the baseline examination with the participants in light indoor clothing without shoes. Body mass index (BMI) was calculated as kilograms per meters squared.

Salt sensitivity phenotypes were defined as the absolute changes in SBP, DBP, and MAP from baseline to low-sodium intervention and from low-sodium intervention to high-sodium intervention. The means of 9 BP measures at baseline and on days 5, 6, and 7 of each intervention stage were calculated. Mean BP responses to low-sodium intake were calculated as the mean BP measures during low-sodium intervention minus the mean BP at baseline, and mean BP responses to high-sodium intake as mean BP during high-sodium intervention minus that during low-sodium intervention.

### Single Nucleotide Polymorphism (SNP) Genotyping of *SGK* Genes

SNPs located within the *SGK* genes and their ±5,000 base-pair flanking regions were genotyped among all participants using chip based hybridization assays (Affymetrix 6.0, Santa Clara, CA). SNPs were excluded if they had a call rate less than 95%, were not in Hardy-Weinberg Equilibrium after adjustment for multiple comparisons (Bonferroni correction), or had a minor allele frequency (MAF) less than 1%. MapMaker/Sibs and PedCheck were used to check for Mendelian inconsistencies within families for each marker. Among the 71 SNPs within *SGK* genes, 56 met the quality control criteria, and 39 were tagged (r^2^<0.9) using Haploview software for inclusion in the current analysis [22]. Detailed information about the *SGK* genes and the 39 SNPs including their genomic locations, MAFs, call rates and *P* values for HWE tests are presented in **Table 1** and **Table S1**.

**Table 1:** Characteristics of *SGK1, SGK2* and *SGK3* genes.

Genesymbol	Gene name	Locus	Physical position±5,000 bp	Tag-SNPs	Function
*SGK1*	serum/glucocorticoid regulated kinase 1	6q23	(134485384,134644196)	29	Contributes to Na^+^ retention and K^+^elimination of the kidney.
*SGK2*	serum/glucocorticoid regulated kinase 2	20q13.2	(42182635,42219273)	8	Encodes a serine/threonine protein kinase.
*SGK3*	serum/glucocorticoid regulated kinasefamily, member 3	8q12	(67619653,67779257)	2	Involved in neutral amino acid transport andactivation of potassium and chloride channels.

Chr, chromosome; SNP, single-nucleotide polymorphism.

### Statistical Analysis

The means or percent of baseline characteristics and BP response variables were calculated for all participants. Additive associations between single SNPs and BP responses to each sodium intervention were assessed using a mixed linear regression model to account for familial correlations. Age, gender, field center, and BMI were controlled in all the association analyses. To correct for multiple testing with the 39 variants, the Bonferroni method was used (α-threshold = 0.05/39 = 1.28×10^−3^). For significant SNPs, we estimated the mean BP responses and 95% confidence interval (CI) for each genotype using a mixed linear regression model. The association analyses were conducted using SAS software (version 9.2; SAS Institute, Inc., Cary, North Carolina).

The joint effect of variants in the *SGK* genes on BP responses to sodium intervention was evaluated by combining *P* values from single SNP association analyses using the truncated product method (TPM) [23], [24]. Truncation point was set as τ = 0.10, and the *P* value for TPM was estimated by 10,000 simulations. If 10,000 simulations failed to generate a *P* value, simulations were increased up to 1,000,000. Sensitivity analyses were conducted using TPM after excluding significant SNPs within a gene to examine their influence on the gene-based analysis. Bonferroni correction was applied to account for multiple testing with 3 genes (α-threshold = 0.05/3 = 0.017). Gene-based analysis was performed using R software (Version 2.15.2; http://www.r-project.org).

## Results

The GenSalt participant’s baseline characteristics and BP responses to sodium interventions are shown in **Table 2**. Blood pressure significantly decreased in response to the low-sodium dietary intervention and increased in response to the high-sodium dietary intervention (all P<0.0001). In response to the low-sodium intervention, decreases of 5.5 mmHg, 2.8 mmHg, and 3.7 mmHg were observed for SBP, DBP and MAP, respectively. SBP, DBP and MAP increased 4.9 mmHg, 1.9 mmHg, and 2.9 mmHg, respectively, in response to high-sodium intervention.

**Table 2:** Characteristics of 1,906 GenSalt dietary intervention participants.

Variables	Mean SD or percentage	Median (interquartile range)
Age	38.7±9.6	39.0 (33.0 to 46.0)
Men, %	53.0	
BMI, kg/m^2^	23.3±3.2	22.9 (21.1 to 25.2)
Creatinine level at baseline, mg/dL	0.9 (0.2)	0.9 (0.8 to 1.1)
Baseline blood pressure		
SBP	116.9±14.2	115.8 (106.4 to 127.1)
DBP	73.7±10.3	73.3 (66.7 to 80.7)
MAP	88.1±10.9	87.7 (80.1 to 95.4)
Response to low sodium intervention		
SBP, mm Hg	−5.5±7.0*	−4.4 (−8.9 to −1.3)
DBP, mm Hg	−2.8±5.5*	−2.7 (−5.6 to 0.4)
MAP, mm Hg	−3.7±5.3*	−3.3 (−6.6 to −0.6)
Response to high sodium intervention		
SBP, mm Hg	4.9±6.0*	4.4 (0.6 to 8.2)
DBP, mm Hg	1.9±5.4*	1.8 (−1.6 to 5.3)
MAP, mm Hg	2.9±5.0*	2.7 (−0.4 to 5.9)

BMI, body mass index; SBP, systolic blood pressure; DBP, diastolic blood pressure; MAP, mean arterial pressure;

*P<0.0001 when comparing blood pressure change with 0.

**Figure 1:**
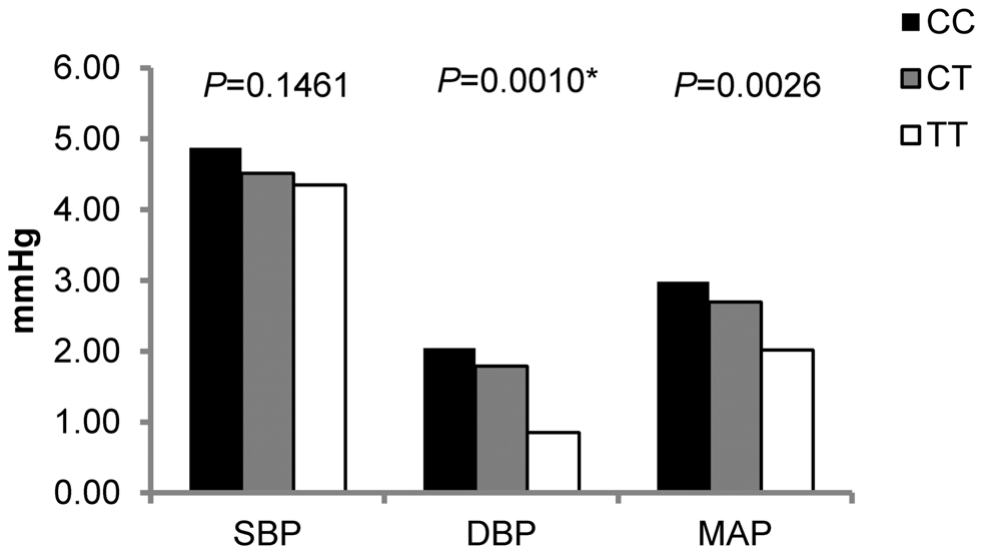
–log_10_ *P* values for the associations of 39 SNPs in *SGK1*, *SGK2* and *SGK3* with blood pressure responses to low-sodium intervention (a) and high-sodium intervention (b). Labeled SNP was significant after Bonferroni correction. The horizontal dashed lines indicate the Bonferroni corrected significant level.

**Figure 2:**
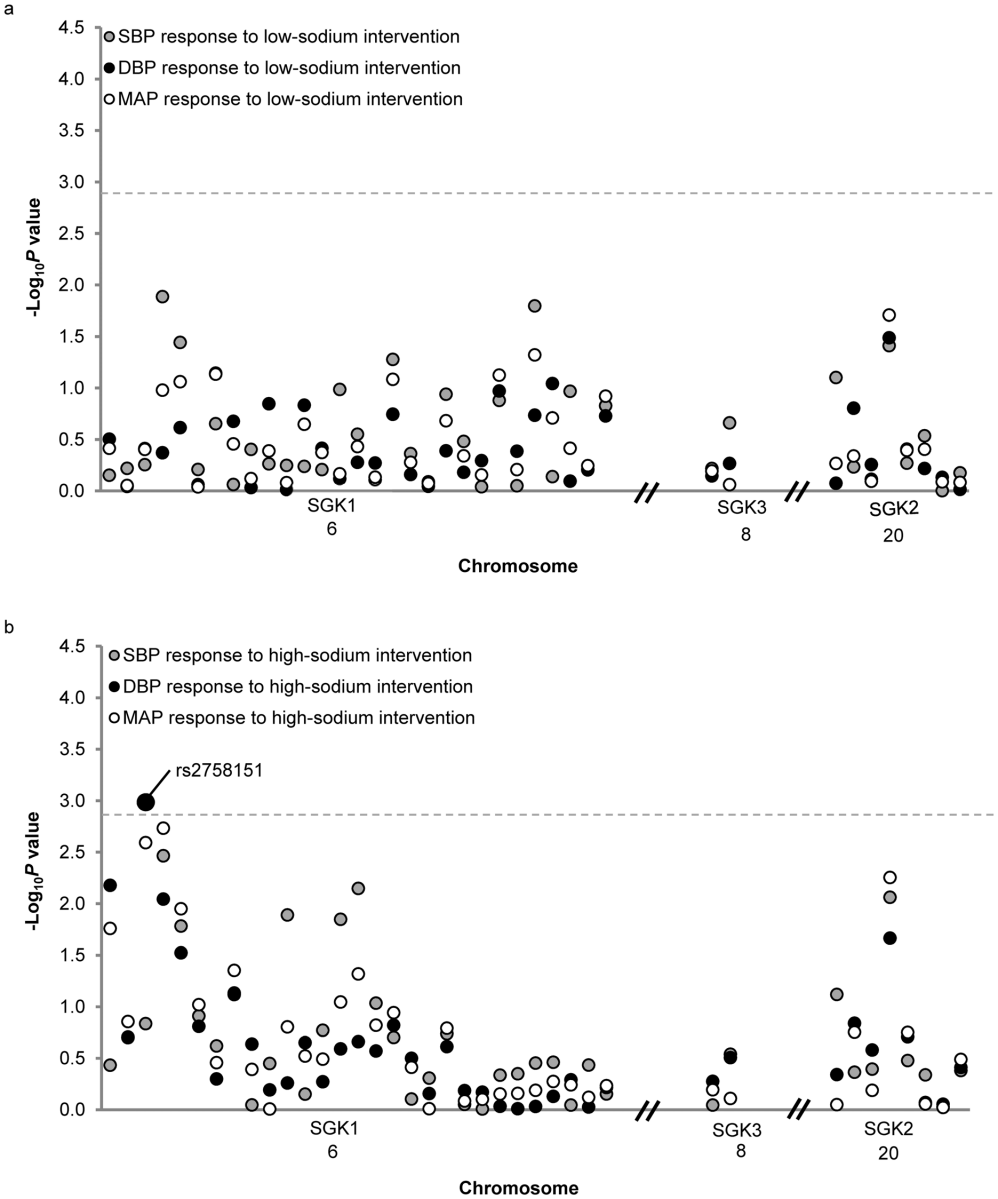
Blood pressure responses to high sodium intervention among participants with different genotypes of rs2758151, respectively. *significant after Bonferroni correction.

The single-marker association analyses are shown in **Figure 1**. SNP, rs2758151, which is located 2.7 kbp downstream to the *SGK1* gene, was significantly associated with absolute DBP responses to high-sodium intervention (*P* = 0.001). As shown in **Figure 2**, DBP responses to high-sodium intervention decreased with the number of minor T alleles of rs2758151. Mean DBP response was 2.04 (1.57 to 2.52) mmHg for participants with CC genotype (n = 510), 1.79 (1.42 to 2.16) mmHg for those with CT genotype (n = 911), and 0.85 (0.30 to 1.41) mmHg for those with TT genotype (n = 415). Similar trends were observed for SBP and MAP response to the high sodium interventions (*P* = 0.146 and 0.003, respectively). Exact *P* values for all single SNP association tests are shown in **Table S2**.

**Table 3** shows the results of the gene-based analyses. *SGK1* was significantly associated with SBP, DBP and MAP responses to high-sodium intervention. In sensitivity analyses excluding significant *SGK1* marker rs2758151, all statistically significant *SGK1* results remained significant with the exception of DBP response to high-sodium. *P*-values for the association between *SGK1* and SBP, DBP, and MAP responses in the sensitivity analysis were 9.0×10^−5^, 0.09 and 5.2×10^−4^, respectively.

**Table 3:** Gene-based associations of *SGK1*, *SGK2* and *SGK3* with blood pressure responses to dietary sodium intervention.

	*SGK1*	*SGK2*	*SGK3*
Response to low-sodium intervention			
SBP	0.1541	0.1037	0.1708
DBP	0.6240	0.3212	0.1326
MAP	0.2474	0.2589	0.1505
Response to high-sodium intervention			
SBP	0.0002*	0.0422	0.2008
DBP	0.0076*	0.2669	0.1388
MAP	1.00E-05*	0.1822	0.1309

BP, blood pressure; SBP, systolic blood pressure; DBP, diastolic blood pressure; MAP, mean arterial pressure;

*Significant *P* values after Bonferroni correction.

## Discussion

In the first study to examine the association between the *SGK* gene pathway and salt-sensitivity of BP in an East Asian population, we identified a significant association between *SGK1* variant rs2758151 and DBP response to high-sodium intervention. Each copy of the rs27858151 minor T allele predicted smaller DBP responses to the high-sodium diet. Similar trends were observed for the SBP and MAP responses to high sodium phenotypes although not significant. In addition, gene-based analysis revealed that the *SGK1* gene was significantly associated with all BP responses to the high-sodium intervention. While it appeared that the gene-based association of the DBP response phenotype could be explained by significant marker rs27858151, the SBP and MAP response phenotypes remained significant after sensitivity analyses excluding this SNP. Neither the gene-based nor single-marker analyses identified significant association of the *SGK2* and *SGK3* genes with the BP response phenotypes. To the best of our knowledge, this is the first study examining the joint effect of SNPs in genes of the SGK family on the blood pressure salt-sensitivity phenotype. In aggregate, these findings could provide valuable information towards delineating the genomic mechanisms underlying human BP salt-sensitivity.

*SGK1* variant rs2758151 showed evidence of association with BP salt-sensitivity in the current analysis. It is of interest to note that this variant was implicated in the only other candidate gene study to examine the relationship between *SGK1* and BP salt-sensitivity to date [17]. In the previous report, Rao and colleagues identified a significant association between rs2758151 and BP response to high sodium intervention in a population of European ancestry [17]. However, their study reported an opposite effect direction of the T allele [17]. A potential reason for this discrepancy could be that rs2758151 is not the causal locus for the salt-sensitivity phenotype but is in linkage disequilibrium (LD) with the causal variant [25]. The causal variant may be on different haplotypes in populations of distinct ethnicities. In support of this hypothesis, post-hoc comparison of LD patterns between European and Chinese populations at the *SGK1* locus showed distinct differences in LD structure between these populations (*P* = 0.01 for differences in LD pattern; see **Figure S1**) [26], [27]. In addition, differences in direction of association could be due to interactive effects with other variants or environmental factors that vary between populations [28]. Lin et al used theoretical modeling to demonstrate that such “flip-flop” associations may indeed represent confirmations of true associations due to interactive effects or LD with a causal variant at another locus [29]. Marker rs2758151 is located downstream of the *SGK1* gene and is unlikely to play a causal role in altering the synthesis or structure of the SGK1 protein. Future studies will be needed to pinpoint the true functional variant responsible for the strong signal reported by both us and Rao et al. Such work could leverage LD information in East Asians and Caucasians to help localize the region for sequencing study.

Our study also represents the first report of gene-based associations of *SGK1* and BP salt-sensitivity phenotypes. We identified significant associations of *SGK1* with SBP, DBP and MAP responses to high-sodium intervention. Although sensitivity analyses revealed that the association between *SGK1* and DBP response was largely driven by marker rs2758151, this variant could not explain the gene-based relation of *SGK1* with both SBP and MAP responses to the high-sodium dietary intervention. Such findings indicate that there might be multiple independent loci within *SGK1* that are related to these phenotypes. Furthermore, our findings highlight the importance of considering both singular and joint effects of variants to elucidate the genetic architecture of complex phenotypes like BP response to dietary sodium intervention.

*SGK2* and *SGK3* were not associated with BP salt-sensitivity in the current analysis, nor were any single variants within the two genes. Although very few studies have been conducted to examine the association of the *SGK2* and *SGK3* genes with BP phenotypes [8], [30], our results are consistent with animal models. For example, Schnackenberg and colleagues showed that *SGK2* knockout mice did not exhibit urinary Na^+^ wasting or low blood pressure [31]. Similarly, *SGK3* knockout mice were not found to have obvious defects in Na^+^ handling in a manuscript by McCormick and collagues [32].

Our study has several important strengths. GenSalt is the largest dietary intervention study to examine the associations between the *SGK* gene pathway and BP responses to dietary sodium intervention. A high proportion of study participants completed the dietary intervention (97.6%), and compliance with the study interventions was excellent, as confirmed by urinary excretion of sodium and potassium during each intervention period. Measurement error was reduced and power enhanced by the large number of BP measures that were collected for each participant. Furthermore, stringent quality control methods were used in measuring BP and other study covariables, genotyping, and marker data cleaning. Finally, the recruitment of only Han Chinese participants should make the analysis robust to even fine levels of population stratification. Although the Affymetrix 6.0 platform generally provides good genomic coverage of common variants in the Han Chinese population [33], coverage of *SGK3* was limited, with only two tag SNPs in the *SGK3* gene. Therefore, common *SGK3* variants associated with BP salt-sensitivity may have been missed by the current study. Further, the gene-based analysis for *SGK3* must be interpreted with caution. Finally, due to the uniqueness of salt-sensitivity phenotype, our findings could not be replicated in an independent East Asian sample.

The current study identified a significant association of *SGK1* marker rs2758151 with BP salt-sensitivity. Having previously been reported to associate with BP responses to dietary sodium in a population of European ancestry, these findings provide some evidence of trans-ethnic replication in the Han Chinese population. Furthermore, our gene-based analysis revealed potentially important joint actions of *SGK1* SNPs on the salt-sensitivity phenotypes. These findings contribute important information towards elucidating the genomic mechanisms underlying blood pressure regulation. Still, further studies will be necessary to localize the reported *SGK1* signals and identify the causal variants for BP salt sensitivity.

## Supporting Information

Figure S1 (TIF)

Table S1 **Quality control information of the tagged 39 SNPs in** ***SGK1*** **,** ***SGK2*** **and** ***SGK3.***
(DOC)

Table S2 ***P*** **-Values of single SNP association analysis of the 39 SNPs in** ***SGK1*** **,** ***SGK2*** **and** ***SGK3*** **with blood pressure responses to dietary sodium intervention.**
(DOCX)
