# Supplementary material for: A Gene-Based Analysis of Variants in the Serum/Glucocorticoid Regulated Kinase (SGK) Genes with Blood Pressure Responses to Sodium Intake: The GenSalt Study
Source: PLoS One. 2014 May 30;9(5):e98432. doi: 10.1371/journal.pone.0098432 (PMC4039502; doi:10.1371/journal.pone.0098432)
Supplement: Table S1 — Quality control information of the tagged 39 SNPs in SGK1 , SGK2 and SGK3. (DOC) [file pone.0098432.s002.doc]

*Table S1. Quality control information of the tagged 39 SNPs in SGK1, SGK2 and SGK3*

| Gene (locus) | SNP | Chromosome | Physical Location | Alleles (Major/Minor) | MAF | Genotyping Call Rate (%) | HW  *P*-Value |
| --- | --- | --- | --- | --- | --- | --- | --- |
| *SGK1* | rs12663728 | 6 | 134528818 | T:C | 0.47 | 99.8 | 0.97 |
| (6q23) | rs2758150 | 6 | 134528907 | G:A | 0.06 | 99.9 | 0.06 |
|  | rs2758151 | 6 | 134529361 | C:T | 0.47 | 99.8 | 0.57 |
|  | rs1057293 | 6 | 134535090 | G:A | 0.16 | 100 | 0.03 |
|  | rs1743966 | 6 | 134535640 | A:G | 0.21 | 99.9 | 0.06 |
|  | rs1763528 | 6 | 134543920 | A:C | 0.36 | 99.9 | 0.01 |
|  | rs1075427 | 6 | 134545897 | G:A | 0.11 | 99.9 | 0.95 |
|  | rs9389148 | 6 | 134557805 | C:G | 0.38 | 100 | 0.41 |
|  | rs6569934 | 6 | 134567898 | C:T | 0.19 | 100 | 0.32 |
|  | rs9493858 | 6 | 134576852 | G:A | 0.07 | 99.7 | 0.10 |
|  | rs17053580 | 6 | 134581647 | G:A | 0.19 | 99.9 | 0.37 |
|  | rs1763496 | 6 | 134587898 | C:T | 0.03 | 99.9 | 0.62 |
|  | rs1009840 | 6 | 134588378 | A:G | 0.27 | 100 | 0.95 |
|  | rs9493867 | 6 | 134589836 | A:T | 0.04 | 100 | 0.52 |
|  | rs1743938 | 6 | 134602439 | A:G | 0.21 | 100 | 0.84 |
|  | rs9493871 | 6 | 134606559 | C:A | 0.05 | 100 | 0.28 |
|  | rs9373086 | 6 | 134612871 | G:T | 0.45 | 100 | 0.23 |
|  | rs9493873 | 6 | 134618204 | A:G | 0.10 | 100 | 0.21 |
|  | rs6924468 | 6 | 134621907 | G:A | 0.14 | 98.2 | 0.66 |
|  | rs4896035 | 6 | 134624037 | T:C | 0.31 | 99.7 | 0.96 |
|  | rs13437143 | 6 | 134637293 | A:T | 0.23 | 100 | 0.20 |
|  | rs9376026 | 6 | 134644147 | C:T | 0.18 | 99.9 | 0.89 |
|  | rs9373088 | 6 | 134651674 | T:A | 0.36 | 100 | 0.43 |
|  | rs6569936 | 6 | 134656194 | A:T | 0.06 | 99.9 | 1.00 |
|  | rs9389154 | 6 | 134656454 | T:C | 0.45 | 100 | 0.05 |
|  | rs6569937 | 6 | 134662366 | G:A | 0.07 | 96.9 | 0.11 |
|  | rs17053584 | 6 | 134664207 | C:G | 0.08 | 99.9 | 0.05 |
|  | rs9493897 | 6 | 134672476 | G:A | 0.25 | 100 | 0.66 |
|  | rs9402588 | 6 | 134672505 | A:G | 0.40 | 100 | 0.68 |
| *SGK3* | rs16933043 | 8 | 67815357 | C:G | 0.02 | 96.5 | 1.00 |
| (8q12) | rs16933080 | 8 | 67922822 | C:T | 0.02 | 100 | 0.76 |
| *SGK2* | rs6093854 | 20 | 41616316 | A:T | 0.10 | 99.8 | 0.47 |
| (20q13.2) | rs743998 | 20 | 41618950 | C:T | 0.19 | 99.2 | 0.08 |
|  | rs3752558 | 20 | 41621277 | A:G | 0.05 | 100 | 0.98 |
|  | rs2071973 | 20 | 41629503 | A:G | 0.24 | 99.7 | 0.42 |
|  | rs2067061 | 20 | 41629964 | A:C | 0.48 | 98.9 | 0.06 |
|  | rs3127061 | 20 | 41630837 | A:C | 0.04 | 99.8 | 0.03 |
|  | rs916410 | 20 | 41636651 | A:G | 0.28 | 99 | 0.61 |
|  | rs1894668 | 20 | 41652333 | C:G | 0.20 | 100 | 0.84 |

SNP, single nucleotide polymorphism; MAF, minor allele frequency; HW, Hardy Weinberg
