# Supplementary material for: A Gene-Based Analysis of Variants in the Serum/Glucocorticoid Regulated Kinase (SGK) Genes with Blood Pressure Responses to Sodium Intake: The GenSalt Study
Source: PLoS One. 2014 May 30;9(5):e98432. doi: 10.1371/journal.pone.0098432 (PMC4039502; doi:10.1371/journal.pone.0098432)
Supplement: Table S2 — P -Values of single SNP association analysis of the 39 SNPs in SGK1 , SGK2 and SGK3 with blood pressure responses to dietary sodium intervention. (DOCX) [file pone.0098432.s003.docx]

Table S2. *P*-Values of single SNP association analysis of the 39 SNPs in *SGK1*, *SGK2* and *SGK3* with blood pressure responses to dietary sodium intervention

| Gene (locus) | SNP | Chr | Physical Position | Absolute Blood Pressure Response | | | | | | |
| --- | --- | --- | --- | --- | --- | --- | --- | --- | --- | --- |
|  |  |  |  | Low-sodium intervention | | |  | High-sodium intervention | | |
|  |  |  |  | SBP | DBP | MAP |  | SBP | DBP | MAP |
| *SGK1* | rs12663728 | 6 | 134528818 | 0.7042 | 0.3137 | 0.3853 |  | 0.3693 | 0.0066 | 0.0174 |
| 6q23 | rs2758150 | 6 | 134528907 | 0.6026 | 0.9063 | 0.8858 |  | 0.1993 | 0.1973 | 0.1393 |
|  | rs2758151 | 6 | 134529361 | 0.5567 | 0.3869 | 0.3946 |  | 0.1461 | **0.0010**^€^ | 0.0026 |
|  | rs1057293 | 6 | 134535090 | 0.0130 | 0.4262 | 0.1052 |  | 0.0034 | 0.0090 | 0.0019 |
|  | rs1743966 | 6 | 134535640 | 0.0361 | 0.2423 | 0.0870 |  | 0.0165 | 0.0300 | 0.0112 |
|  | rs1763528 | 6 | 134543920 | 0.6187 | 0.8688 | 0.9140 |  | 0.1231 | 0.1552 | 0.0957 |
|  | rs1075427 | 6 | 134545897 | 0.2221 | 0.0718 | 0.0733 |  | 0.2400 | 0.5021 | 0.3486 |
|  | rs9389148 | 6 | 134557805 | 0.8668 | 0.2103 | 0.3491 |  | 0.0737 | 0.0761 | 0.0444 |
|  | rs6569934 | 6 | 134567898 | 0.3942 | 0.9270 | 0.7584 |  | 0.8991 | 0.2309 | 0.4055 |
|  | rs9493858 | 6 | 134576852 | 0.5454 | 0.1427 | 0.4080 |  | 0.3556 | 0.6417 | 0.9848 |
|  | rs17053580 | 6 | 134581647 | 0.5656 | 0.9711 | 0.8327 |  | 0.0129 | 0.5502 | 0.1572 |
|  | rs1763496 | 6 | 134587898 | 0.5782 | 0.1470 | 0.2250 |  | 0.7061 | 0.2236 | 0.3008 |
|  | rs1009840 | 6 | 134588378 | 0.6211 | 0.3853 | 0.4226 |  | 0.1695 | 0.5361 | 0.3230 |
|  | rs9493867 | 6 | 134589836 | 0.1034 | 0.7605 | 0.6812 |  | 0.0142 | 0.2563 | 0.0900 |
|  | rs1743938 | 6 | 134602439 | 0.2813 | 0.5265 | 0.3710 |  | 0.0071 | 0.2188 | 0.0481 |
|  | rs9493871 | 6 | 134606559 | 0.7816 | 0.5351 | 0.7345 |  | 0.0923 | 0.2693 | 0.1515 |
|  | rs9373086 | 6 | 134612871 | 0.0528 | 0.1801 | 0.0826 |  | 0.1988 | 0.1512 | 0.1144 |
|  | rs9493873 | 6 | 134618204 | 0.4334 | 0.6925 | 0.5271 |  | 0.7890 | 0.3171 | 0.3876 |
|  | rs6924468 | 6 | 134621907 | 0.8165 | 0.9035 | 0.8451 |  | 0.4936 | 0.6967 | 0.9804 |
|  | rs4896035 | 6 | 134624037 | 0.1151 | 0.4070 | 0.2081 |  | 0.1829 | 0.2443 | 0.1618 |
|  | rs13437143 | 6 | 134637293 | 0.3295 | 0.6592 | 0.4556 |  | 0.8882 | 0.6517 | 0.8239 |
|  | rs9376026 | 6 | 134644147 | 0.9104 | 0.5077 | 0.6986 |  | 0.9843 | 0.6752 | 0.7942 |
|  | rs9373088 | 6 | 134651674 | 0.1321 | 0.1069 | 0.0750 |  | 0.4619 | 0.9295 | 0.7030 |
|  | rs6569936 | 6 | 134656194 | 0.8898 | 0.4117 | 0.6207 |  | 0.4475 | 0.9782 | 0.6940 |
|  | rs9389154 | 6 | 134656454 | 0.0160 | 0.1836 | 0.0477 |  | 0.3528 | 0.9316 | 0.6477 |
|  | rs6569937 | 6 | 134662366 | 0.7260 | 0.0906 | 0.1949 |  | 0.3461 | 0.7420 | 0.5318 |
|  | rs17053584 | 6 | 134664207 | 0.1078 | 0.8031 | 0.3845 |  | 0.9028 | 0.5125 | 0.5736 |
|  | rs9493897 | 6 | 134672476 | 0.6238 | 0.6160 | 0.5684 |  | 0.3684 | 0.9441 | 0.7591 |
|  | rs9402588 | 6 | 134672505 | 0.1492 | 0.1868 | 0.1202 |  | 0.7033 | 0.6083 | 0.5839 |
| *SGK3* | rs16933043 | 8 | 67815357 | 0.6051 | 0.7148 | 0.6414 |  | 0.9019 | 0.5302 | 0.6409 |
| 8q12 | rs16933080 | 8 | 67922822 | 0.2178 | 0.5398 | 0.8678 |  | 0.2892 | 0.3120 | 0.7782 |
| *SGK2* | rs6093854 | 20 | 41616316 | 0.0791 | 0.8410 | 0.5397 |  | 0.0761 | 0.4554 | 0.8928 |
| 20q13.2 | rs743998 | 20 | 41618950 | 0.5850 | 0.1569 | 0.4582 |  | 0.4324 | 0.1450 | 0.1766 |
|  | rs3752558 | 20 | 41621277 | 0.7720 | 0.5540 | 0.8055 |  | 0.4033 | 0.2631 | 0.6489 |
|  | rs2071973 | 20 | 41629503 | 0.0388 | 0.0326 | 0.0196 |  | 0.0087 | 0.0216 | 0.0056 |
|  | rs2067061 | 20 | 41629964 | 0.5364 | 0.3928 | 0.4033 |  | 0.3326 | 0.1957 | 0.1778 |
|  | rs3127061 | 20 | 41630837 | 0.2908 | 0.6053 | 0.3934 |  | 0.4589 | 0.8521 | 0.8809 |
|  | rs916410 | 20 | 41636651 | 0.9931 | 0.7386 | 0.8169 |  | 0.9337 | 0.8864 | 0.9514 |
|  | rs1894668 | 20 | 41652333 | 0.6675 | 0.9667 | 0.8263 |  | 0.4163 | 0.3883 | 0.3245 |

SNP, single-nucleotide polymorphism; SBP, systolic blood pressure; DBP, diastolic blood pressure; MAP, mean arterial pressure;

All analyses were controlling for age, gender, field center, and body mass index;

^€^Significant after Bonferroni Correction;
